# Hedgehog Signaling and Truncated GLI1 in Cancer

**DOI:** 10.3390/cells9092114

**Published:** 2020-09-17

**Authors:** Daniel Doheny, Sara G. Manore, Grace L. Wong, Hui-Wen Lo

**Affiliations:** 1 Department of Cancer Biology, Wake Forest University School of Medicine, Winston-Salem, NC 27101, USA; ddoheny@wakehealth.edu (D.D.); smanore@wakehealth.edu (S.G.M.); glwong@wakehealth.edu (G.L.W.); 2 Wake Forest Comprehensive Cancer Center, Wake Forest University School of Medicine, Winston-Salem, NC 27101, USA

**Keywords:** hedgehog signaling, tumor–stroma interaction, development, target therapy, tGLI1

## Abstract

The hedgehog (HH) signaling pathway regulates normal cell growth and differentiation. As a consequence of improper control, aberrant HH signaling results in tumorigenesis and supports aggressive phenotypes of human cancers, such as neoplastic transformation, tumor progression, metastasis, and drug resistance. Canonical activation of HH signaling occurs through binding of HH ligands to the transmembrane receptor Patched 1 (PTCH1), which derepresses the transmembrane G protein-coupled receptor Smoothened (SMO). Consequently, the glioma-associated oncogene homolog 1 (GLI1) zinc-finger transcription factors, the terminal effectors of the HH pathway, are released from suppressor of fused (SUFU)-mediated cytoplasmic sequestration, permitting nuclear translocation and activation of target genes. Aberrant activation of this pathway has been implicated in several cancer types, including medulloblastoma, rhabdomyosarcoma, basal cell carcinoma, glioblastoma, and cancers of lung, colon, stomach, pancreas, ovarian, and breast. Therefore, several components of the HH pathway are under investigation for targeted cancer therapy, particularly GLI1 and SMO. GLI1 transcripts are reported to undergo alternative splicing to produce truncated variants: loss-of-function GLI1ΔN and gain-of-function truncated GLI1 (tGLI1). This review covers the biochemical steps necessary for propagation of the HH activating signal and the involvement of aberrant HH signaling in human cancers, with a highlight on the tumor-specific gain-of-function tGLI1 isoform.

## 1. Introduction

The hedgehog (HH) gene was first discovered by Christiane Nusslein-Volhard and Eric F. Weischaus in 1980 through a screen for embryonic lethal mutants of *Drosophila melanogaster* that disrupted the larval body plan [1]. Aptly named for the short, “spiked” phenotype of the cuticle of the mutant *Drosophila* larvae, HH signaling is evolutionarily conserved from flies to humans and has been deemed a key regulator of several fundamental processes in vertebrate embryonic development including cell fate, patterning, proliferation, survival, and differentiation [2,3]. HH signals are diverse and contextually dependent. For instance, HH signals may act as inducing factors controlling the form of developing organs or as dose-dependent morphogens that cause distinct cell fates in a particular target field [3]. The importance of HH signaling in the development of higher-order organisms is clearly depicted by the unfortunate phenotypic consequences in human fetuses. Dysregulated HH signaling commonly results in fetuses with brain, facial, and other midline deficiencies including holoprosencephaly (lack of forebrain development), microencephaly, and cyclopia (an extreme form of holoprosencephaly characterized by failure of the embryonic prosencephalon to cleave into the left and right hemispheres) [4,5,6]. Likewise, hyperactive HH signaling is not without consequence in adults.

Aberrant HH signaling has been recognized as one of the important signaling pathways and viable therapeutic targets in human cancers. Continuous HH pathway activation plays a pathological role in the growth of a group of endodermally-derived human cancers that account for ~25% of human cancer [7,8]. Under normal conditions, the activation of the HH pathway is limited to stem cell subpopulations that undergo rapid turnover to maintain homeostasis via modulating tissue repair of the intestines, nervous system, and skin [9,10,11,12]. In adults, mutation or dysregulation of this signaling axis plays a key role in proliferation and differentiation that promotes tumorigenesis and tumor growth in several tissue types. Deregulated HH signaling is typified by basal cell carcinoma (BCC) and medulloblastoma, two well-recognized cancers with protein mutations of HH pathway components [13,14,15]. In addition, dysregulation of HH signaling activity has also been linked to the pathologies of breast, lung, pancreas, and prostate cancers [4,16,17,18]. Due to the importance of HH signaling in human cancer, attempts to therapeutically target this pathway have been extensive, which have resulted in three US FDA-approved inhibitors available for use in cancers to date: arsenic trioxide, vismodegib/GDC-0449, and sonidegib/LDE-225 [19].

**Figure 1:**
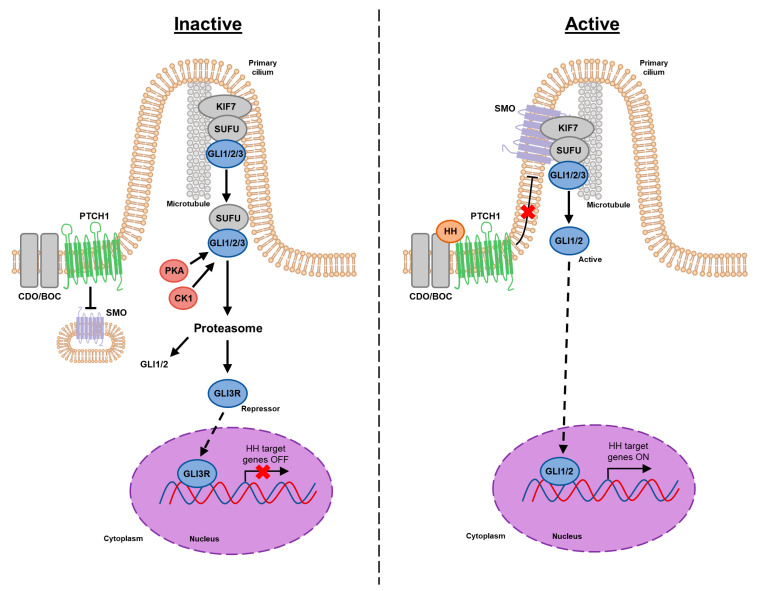
Overview of the hedgehog signaling pathway. Inactive signaling (**left**) occurs in the absence of HH ligand in which PTCH1 precludes SMO localization to the primary cilium, leading to GLI1 sequestration in the cytoplasm by SUFU. GLI proteins are then marked for processing, e.g., through PKA- or CK1-dependent phosphorylation, and processed into transcriptional repressors (GLI3R). Upon CDO/BOC-mediated binding of HH ligand to PTCH1 (**right**), SMO is derepressed and localizes to the primary cilium. A protein complex containing KIF7 and SUFU bound to GLI transcription factors is dynamically trafficked to the activated SMO. Active SMO promotes release of GLI proteins from SUFU, resulting in nuclear accumulation of GLI1/2 and activation of target genes.

Canonical signaling is initiated by HH ligand binding to the transmembrane Patched 1 (PTCH1) receptor (Figure 1) [20]. SMO activity is constitutively repressed by PTCH1; however, this inhibition is abrogated upon ligand binding to PTCH1. Activated SMO relays the HH activating signals into the cytoplasm, which ultimately releases GLI transcription factors from cytoplasmic sequestration by suppressor of fused (SUFU), thereby permitting GLI to translocate into the nucleus and bind transcriptional targets to regulate cellular gene expression [20,21]. There are three GLI isoforms in mammals which act as the terminal effectors of the HH pathway: (1) GLI1 typically induces gene expression and is thought to be a reliable biomarker of pathway activation, (2) GLI12 can either induce or repress gene expression, and (3) GLI3 acts as a transcriptional repressor in most contexts [22]. Interestingly, *GLI1* somatic mutations have not been reported as a cause of aberrant signaling. In contrast, several other pathway components present with mutations, including *HH*, *PTCH1*, *SMO*, and *SUFU*. However, recent evidence indicates that the GLI1 mRNA transcript can undergo alternative splicing, leading to the synthesis of an N-terminal deletion variant (GLI1ΔN) and truncated GLI1 variant (tGLI1) discovered in our laboratory [23,24]. Research is currently underway to determine the importance of these splice variants compared to the full-length GLI1. GLIΔN appears to function similarly to the wild-type GLI1, albeit with weaker gene activation, and is expressed at equal or lower levels in cancers compared to normal tissues [23]. In stark contrast, the tGLI1 variant has been shown to be frequently expressed in several cancers including glioblastoma, breast, and liver cancer, but not in normal tissues or cells [24,25,26,27]. Furthermore, tGLI1 is a gain-of-function transcription factor that, in addition to regulating known GLI1 target genes, is able to modulate expression of genes not regulated by GLI1 to induce several oncogenic phenotypes in breast cancer and glioblastoma such as tumor growth, migration and invasion, angiogenesis, maintenance of the cancer stem cell (CSC) population, metastasis, and astrocyte activation [24,25,28,29,30,31,32,33]. These data strongly suggest that tGLI1 could be a more potent transcriptional regulator than GLI1 or GLI1ΔN. In light of these new findings, this review summarizes the modes of HH signaling, the structures and properties of the three GLI1 isoforms, and the role of aberrant HH activation in cancer.

## 2. Canonical HH Signaling

The canonical mammalian pathway includes several key components including the hedgehog glycoproteins Sonic hedgehog (SHH), Desert hedgehog (DHH), and Indian hedgehog (IHH); the 12-transmembrane protein Patched1 (PTCH1), the 7-transmembrane protein Smoothened (SMO), suppressor of fused (SUFU), and the GLI zinc-finger transcription factors, which act as the terminal effectors of the pathway. As previously stated, the family of GLI transcription factors consists of three separate proteins—GLI1 primarily functions as a transcriptional activator, GLI2 as either an activator or repressor, and GLI3 as a transcriptional repressor [22]. The expression of GLI1 is highly dependent upon active HH signaling and is used as a readout of pathway activation. Of the three HH glycoproteins, SHH, the focus of this review, is the most broadly expressed HH protein and the most potent of the three [2]. Prior to secretion, SHH undergoes several processing steps including removal of its signaling sequence, cleavage catalyzed by its own C-terminal protease domain [34], addition of cholesterol to the C-terminal domain to facilitate association with the plasma membrane [35], and finally the addition of a palmitate at the N-terminal domain which forms the fully active SHH signaling molecule [36,37]. SHH binding is then facilitated by the cell surface Ig/fibronectin superfamily proteins CDO and BOC which directly bind SHH through a specific fibronectin repeat, thereby increasing the affinity of SHH to its signaling receptor PTCH1 [38]. Tukachinsky et al. (2016) recently identified a critical interaction between the palmitoylated N-terminal portion of SHH and PTCH1, and further demonstrated that it is necessary and sufficient for PTCH1 inhibition [39]. Specifically, a short palmitoylated N-terminal SHH peptide was sufficient to bind PTCH1 and activate signaling. The remaining portion of the SHH molecule was found to bind PTCH1 at a separate site, leading to its internalization.

In the absence of SHH ligand, PTCH1 constitutively represses SMO activity [20]. Current literature suggests that this repression is mediated in at least two ways: (1) disruption of SMO localization and (2) catalytic inhibition of SMO [20,40]. In mammalian cells, SMO-dependent GLI1 activation is contingent upon the localization of SMO to the primary cilium, a solitary cell surface projection that functions as a major signaling center. PTCH1 localizes at the base of the primary cilium, precluding accumulation of SMO and thereby impacting its activation [40,41,42]. Binding of SHH to PTCH1 delocalizes PTCH1 from the primary cilium, leading to its degradation and the reciprocal movement of SMO into the cilium. The precise mechanism by which PTCH1 catalytically inhibits SMO remains unknown; however, prevailing models suggest PTCH1 inhibits SMO sub-stoichiometrically in lieu of a direct interaction, by limiting the availability of modulatory ligand(s) to SMO [20,40,42,43]. Cryo-EM structure analysis revealed tetrameric PTCH1 bound to the palmitoylated N-terminal signaling domain of SHH at a 4:2 stoichiometric ratio. The structure shows that four PTCH1 protomers are organized as a loose dimer of dimers in which each dimer binds to one SHH molecule through two distinct inhibitory interfaces [44]. Additionally, PTCH1 shares sequence homology with the prokaryotic resistance-nodulation-division (RND) family transporters, typified by the bacterial proton-driven multidrug resistance exporter AcrB suggesting PTCH1 may act as a SMO ligand transporter [45]. Multiple studies suggest that this endogenous cargo may be a steroidal molecule [46,47,48,49,50]. Importantly, PTCH1 contains a sterol-sensing domain conserved across sterol biogenesis regulatory enzymes likely conferring the ability to bind sterols [51]. For an extensive review on the evidence supporting sterol lipids as the elusive second messenger that communicates the SHH signal between PTCH1 and SMO, we refer readers to a comprehensive article by Kowatsch et al. (2019) [52].

Binding of SHH to the PTCH1 receptor derepresses SMO, ultimately freeing GLI1 transcription factors from cytoplasmic retention. However, the understanding of how the activation signal is transmitted from ciliary SMO to the cytoplasmic GLI1 effectors remains incomplete [53]. As mentioned above, propagation of the SHH signal to GLI1 effectors is dependent upon SMO localization to the primary cilia. SMO is phosphorylated and activated by association with CK1α and GPCR kinase 2 (GRK2), resulting in its translocation to the primary cilium through interaction by β-arrestin [43,54,55]. Evidence has also emerged implicating the cyclic AMP (cAMP)-protein kinase (PKA) pathway in the lateral movement of SMO in mammalian cells [56]. Milenkovic et al. (2009). demonstrated that increasing cAMP levels via Forskolin treatment induced SMO translocation to the primary cilium through a lateral transport pathway [56]. Conversely, inhibition of PKA activity by small-molecule inhibitors H89 and KT5720 abrogated SHH-induced transcription of *PTCH1* and *GLI1* target genes [56]. This novel finding starkly contrasts the canonical description whereby increasing PKA activity inhibits signaling by promoting GLI1 degradation and decreasing PKA activates signaling (even in the absence of SHH ligands) [57,58,59,60,61,62,63,64]. Clearly the involvement of PKA in regulating SHH signaling cannot be distilled down to a binary relationship and additional studies are needed to fully elucidate the impact of PKA on the components of this signaling axis.

Once SMO accumulates in the primary cilia, a protein complex containing kinesin protein (Kif7) and SUFU bound to GLI1 transcription factors is dynamically trafficked to the activated SMO in the primary cilia [65,66,67]. However, there is no evidence that SMO directly interacts with SUFU to release GLI1. Transmission of the activating signal from SMO to SUFU appears to be dependent upon SMO’s interaction with the Ellis van Creveld Syndrome ciliary complex proteins Evc and Evc2 [68,69]. These proteins, which co-associate and localize at the primary cilia in a mutually dependent manner, act downstream of SMO to promote disassociation of SUFU from the GLI proteins. SUFU is essential for suppressing GLI1 activity in unstimulated cells through directly binding GLI1 effectors via an N-terminal interacting site to sequester GLI1 in the cytoplasm [70]. Endogenous SUFU–GLI1 complexes are rapidly dissociated upon recruitment to the primary cilia by activated SMO thus licensing GLI1 proteins for gene transcriptional activation [53,65,71]. GLI1 activation is regulated at several levels via phosphorylation by inhibitors such as SUFU, Ren, protein kinase A (PKA), glycogen synthase kinase 3β (GSK3β) and activators such as Dyrk1, Ras, and Akt [72,73].

## 3. Noncanonical Signaling

The notion of noncanonical SHH signaling refers to the cellular and tissue responses to HH isoforms that are not mediated by GLI1 isoform transcriptional activity. There are three classifications of noncanonical HH signaling: Type I acts through PTCH1 via functions distinct from its inhibition of SMO, Type II is mediated through GLI1-independent activities of SMO such as activation of small GTPases, and Type III refers to all other mechanisms of GLI1 transcription factor activation occurring independently of upstream PTCH1-SMO signaling [74,75].

### 3.1. Type I—PTCH1 Functions Distinct from SMO Inhibition

Canonical SHH signaling combats apoptosis through several mechanisms: upregulation of pro-survival genes (e.g., *Bcl-2*) via GLI1-dependent transcription; activation of Akt, a pro-survival kinase; and the Type I noncanonical pathway [75]. Recent evidence purports PTCH1 to be capable of promoting apoptosis independently of canonical SHH signaling [76,77]. Ectopic expression of PTCH1 induced apoptosis in vitro independent of SMO, as indicated by the finding that SMO overexpression was not able to prevent cell death [77]. PTCH1 is typically described as a tumor suppressor by virtue of its inhibitory action toward SMO. However, the findings by Chinchilla et al. (2010) suggest that the tumor-suppressive capabilities of PTCH1 go beyond its relationship with SMO. Interestingly, PTCH1 has been proposed to function as a dependence receptor in that the function of PTCH1 at the time it is expressed, solely depends on whether the SHH ligand is present [78]. PTCH1 shares several similarities to other dependence receptors [75]. Additionally, the PTCH1 C-terminal domain (CTD), when cleaved, was found to induce cell death and regulate PTCH1 degradation and localization [79]. However, the CTD does not appear necessary for maintaining the fidelity of PTCH1-mediated SMO inhibition [80]. SHH also activates the ERK pathway independently of SMO, most likely through modulating the interaction of the PTCH1 CTD with SH3-containing proteins such as Grb2 [81].

Furthermore, SMO-independent noncanonical signaling can also influence proliferation and cell cycle regulation in some cell types, notably cerebellar granule precursor cells, which may give rise to medulloblastoma through GLI1-mediated induction of *n-myc* and *Cyclin D1* [82,83,84,85,86]. The cyclin protein family acts as regulatory subunits for cyclin-dependent kinases, which are responsible for regulating cell cycle progression by directly affecting a cell’s transcriptional landscape [87]. Specifically involved in traversing the G2/M checkpoint, activated Cyclin B1-Cdk1, essential for mitotic progression, can be regulated by noncanonical SHH signaling [88]. PTCH1 is able to interact with phosphorylated Cyclin B1 through the large intracellular loop between the sixth and seventh transmembrane domains preventing Cyclin B1 from translocating into the nucleus and subsequently reducing proliferation [89]. The identification of Cyclin B1 sequestration by PTCH1 demonstrates a tumor-suppressive function independent of downstream SHH signaling components. As expected, the presence of SHH restores canonical signaling and precludes the PTCH1 interaction with Cyclin B1, permitting its nuclear translocation and completion of mitosis [89]. The multiplicity of PTCH1 tumor-suppressive functions supports *PTCH1* mutations as a strong predictor of breast cancer recurrence meriting further study [16].

### 3.2. Type II—SMO-Mediated Functions Independent of GLI1

SMO, a functional G protein-coupled receptor (GPCR), demonstrates selectivity toward heterotrimeric G_i_ proteins [90]. GPCRs are known to activate small GTPases which play key roles in cytoskeletal reorganization [91]. These small GTPases are monomeric G proteins that act as molecular switches to quickly regulate cellular processes [92]. In the context of SMO-dependent noncanonical SHH signaling, rapid stimulation of Rac1 and RhoA small GTPases by SMO has been identified as a cause of fibroblast migration [93]. In this study, both SHH ligand and purmorphamine, a potent SMO agonist, promote migration of NIH3T3 fibroblasts without affecting SMO-knockout mouse embryonic fibroblasts (MEFs). The reciprocal was also found to be true, i.e., that activation of both G_i_ and PI3K by SMO was required for cell migration and RhoA/Rac1 activation [93]. Despite ectopically expressing GLI3R, the transcriptional repressor of GLI3, neither SHH- nor purmorphamine-mediated MEF migration was disrupted demonstrating the migratory phenotype was mediated by noncanonical signaling [93]. Additional evidence suggests that this form of SMO-independent noncanonical SHH signaling is also independent of localization to the primary cilia, an important step in propagating the activated SHH signal. Ho Wei et al. (2018) found that SHH and purmorphamine-mediated SMO activation stimulated G_i_ proteins and RhoA independently of the presence of primary cilia [94]. Kif3a-deficient fibroblasts, which cannot form primary cilia and therefore activate the canonical SHH pathway, retained the ability to activate RHOA following stimulation of endogenous SMO with SHH or purmorphamine [94].

### 3.3. Type III—Mechanisms Independent of Upstream PTCH1-SMO Signaling

Type III noncanonical hedgehog signaling encompasses all other mechanisms of GLI1 activation that occur independently of the noncanonical PTCH1 and SMO signaling activities described above. This form of GLI1 activation primarily arises through post-translational modification of GLI1 proteins and crosstalk with other oncogenic pathways that promote transcriptional activation of GLI1 genes. GLI1 proteins are extensively modified post-translationally, and these modifications largely determine intracellular trafficking and transcriptional output of the SHH pathway. Known post-translational modifications (PTMs) of GLI1 include phosphorylation, ubiquitination/proteasomal truncation, sumoylation, acetylation, and O-GlcNAcylation [95,96]. Phosphorylation, particularly by protein kinase A (PKA), is the most frequently studied GLI1 PTM. PKA-mediated phosphorylation of GLI1 serine residues is the critical inhibitory signal that limits the conversion of GLI1 proteins into transcriptional activators in the absence of Sonic hedgehog ligand [61]. Casein kinase 1 (CK1) plays a key role in formation of both GLI1 repressor proteins and formation and stabilization of active GLI1 proteins from Cul3/Spop-mediated proteosomal degradation [97]. Dual-specificity tyrosine kinase family (DYRK) proteins are also known to regulate GLI1 proteins through PTM. DYRK1 directly phosphorylates GLI1 enhancing its transcriptional activity in part through retaining GLI1 in the nucleus [98,99,100]. Other kinases known to phosphorylate GLI1 include ULK3 [101,102], S6K1 [103], atypical PKA [104,105,106], AMP-activated protein kinase (AMPK) [107,108,109], Mitogen-activated protein kinase kinase kinase 1 (MEKK1) [110], and Hck [111].

**Figure 2:**
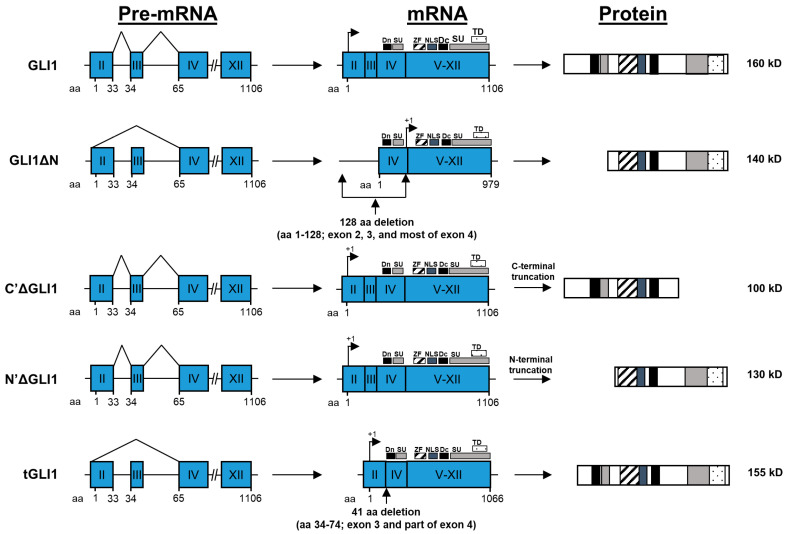
Structures of the GLI1 gene and known GLI1 isoforms. The full-length GLI1 gene consists of 12 exons, including the 5′-untranslated exon I. The GLI1 coding region spans nt +79 to +3399 with the initiating methionine codon, ATG, at +79 in exon II (arrows). Exons are indicated as blue boxes, and introns are shown by lines. The known functional domains of full-length GLI1 include the degron degradation signals (Dn and Dc; aa 77–116; 464–469), SUFU-binding domains (SU; aa 116–125 and C-terminus), zinc-finger domains (ZF; aa 235–387), the nuclear localization signal (NLS; aa 380–420), and the transactivation domain (aa 1020–1091). Alternative splicing of GLI1 RNA can lead to the deletion of exons I–III, totaling 128 amino acids at the N-terminus, forming the GLI1ΔN variant. The C’ΔGLI1 and N’ΔGLI1 variants are proposed to arise from post-translational C-terminal and N-terminal truncations of the full-length GLI1 gene product, respectively. The deletion of exon III and part of exon IV, totaling 41 amino acids, yields the tGLI1 isoform. Importantly, tGLI1 retains all known functional domains of the full-length GLI1. Construct names are on the left and estimated molecular weights are to the right. Protein diagrams are aligned using the zinc-finger domains.

Ubiquitination, a process that targets substrate proteins for proteolytic degradation via the ubiquitin-proteasome system, is an important GLI1 PTM that balances the repressor and active forms of GLI1 to keep the pathway in the off state and acts as a negative feedback look in the on state [112]. Several E3 ligase complexes catalyze the addition of ubiquitin chains to GLI1 lysine residues; two of these have been extensively characterized: Cul1/β-TrCP and E3 ubiquitin-protein ligase Itchy homolog (Itch). Cul1/β-TrCP ubiquitinates GLI1 in its inactive state to induce complete protein degradation [113,114]. This interaction is dependent on the conserved degron motifs (Figure 2) [114]. Conversely, Itch is involved in the degradation of activated GLI1 proteins. Itch-dependent degradation of GLI1 is mediated by the E3 ligase adaptor protein Numb [115,116].

Sumoylation is another repressive modification of many transcription factors that is catalyzed by enzymes similar to ubiquitin ligases. Pias1 small ubiquitin-related modifier (SUMO) ligase was found to decrease GLI1 ubiquitination, thereby increasing GLI1 transcriptional activity in vitro [117]. Alternatively, the sentrin-specific peptidase (SENP1) is the specific deSUMOylation enzyme for GLI1 that stabilizes GLI1 through competition for conserved lysine residues [118]. Furthermore, acetylation of GLI1 at lysine residue K518 by the acetyltransferase p300/CBP reduces the transcriptional activity of GLI1 through sequestering inactive GLI1, inhibition of DNA binding, and increasing nuclear export [105,119]. This repressive marker is further potentiated by β-arrestin1 (Arrb1) [120]. Conversely, histone deacetylase (HDAC) activity has been shown to increase SHH pathway activity [119,121]. Another PTM, O-GlcNAcylation, which is the addition of a single O-linked β-N-acetylglucosamine to specific serine/threonine residues, has recently been shown to increase GLI1 nuclear accumulation and transcriptional activity [122].

## 4. GLI1 Isoforms: Structures and Properties

### 4.1. GLI1

The human *GLI1* gene was identified as a putative oncogene in 1987 by Kinzler et al. after observing the amplification of a region within chromosome 12 [123]. The gene was named by virtue of the glioma tumor in which the amplification was detected. The 3318 base pair sequence codes for a 1106 residue protein that belongs to the GLI-Kruppel family of zinc-finger transcription factors [124,125]. Using a conserved five tandem C2-H2 zinc-finger binding domain (amino acids 234-388), GLI1 functions as a monomer that recognizes the DNA consensus sequence 5′-GACCACCCA-3′ [126]. Only the two cytosine-pairs flanking the central adenine are critical for GLI1 binding; the other positions can tolerate a degree of flexibility if located within natural promoters [127]. Zinc-finger domains two through five bind DNA in the major groove via interactions with the phosphate backbone [128]. To ensure nuclear translocation, GLI1 proteins contain a nuclear export sequence and nuclear localization signal (Figure 2) [129,130]. Furthermore, the GLI1 C-terminal domain (amino acids 1020–1091) confers the ability to transactivate gene promoters through modulation of chromatin remodeling, interaction with histone acetyltransferases, histone deacetylases, SWI-SNF5, and SWI/SNF-like Brg/Brm-associated factor [119,131,132,133]. Other members of the GLI-Kruppel protein family include GLI2 and GLI3. GLI2 is another, albeit less potent, transcriptional activator, while GLI3 primarily represses transcriptional activity in most settings [22,134,135,136]. The remainder of this review will focus on GLI1 and its isoforms.

### 4.2. GLIΔN

Nearly 20 years later, the GLI1 splice variant, GLI1ΔN, was discovered in 2008 by Shimokawa et al. [23]. Skipping of exon II and III results in the loss of the N-terminal degron degradation signal and SUFU-interacting site, the latter of which is responsible for cytoplasmic sequestration of GLI1 (Figure 2) [23,70]. Despite lacking two key N-terminal protein regions involved in tight regulation of GLI1-mediated transcription, luciferase-reporter activation by GLI1ΔN was lower than that of the wild-type GLI1 in NIH3T3 cells [23]. The same held true for the transcriptional response of endogenous genes in the same cells. Furthermore, the N-terminal truncation clearly reduced GLI1ΔN nuclear translocation as noted by the broad diffusion of the protein throughout the cytoplasm and nucleus and punctiform patterning indicative of reduced transcriptional activity, likely due to loss of DYRK1 phosphorylation [23,98]. However, SUFU proved to be a stronger regulator of GLI1 localization compared to GLI1ΔN. One may then speculate that there exists a separate regulatory mechanism controlling the activity of this truncated variant. Indeed, some years later, the same group described a carboxy terminal variant of SUFU, denoted SUFU-ΔC, that was more effective in restricting GLI1ΔN than wild-type GLI1 [21]. Additionally, the observation that GLI1ΔN is downregulated in tumor tissues compared to normal tissues suggests that alternative splicing of GLI1 mRNA may function as a regulatory sink to control pathway activity [23,137]. Unfortunately, the weaker transcriptional activity of GLI1ΔN all but precluded this splice variant as a putative oncogene. Though we may not fully understand the role of GLI1ΔN in SHH signaling, it is clear that alternative splicing is a key biological process that contributes to the diversity of signaling components within the SHH signaling axis.

### 4.3. C’ΔGLI1 and N’ΔGLI1

The GLI1 isoforms C’ΔGLI1 and N’ΔGLI1 were observed following transfection of U251 glioma cells with an N-terminal myc-tagged GLI1 expression plasmid [138]. Using an anti-myc antibody, Western blot analysis revealed the presence of a 110 kD isoform (C’ΔGLI1) in addition to the full-length protein. C’ΔGLI1 presents with a C-terminal truncation, leading to the absence of the C-terminal SUFU-binding region and transactivation domains suggesting this isoform may act as a C’Δ repressor (Figure 2) [63,138]. Moreover, transfection of U87 glioma cells with the same construct produced only trace amounts of C’ΔGLI1 suggesting detection of this isoform was not solely due to ubiquitous degradation [138].

The 130 kD N’ΔGLI1 isoform, distinct from GLI1ΔN, was not discovered until repeat sample analysis using an antibody that targeted a domain immediately C-terminal to the zinc-fingers of the full-length GLI1 protein [138]. The N-terminal truncation removed the myc tag rendering the anti-myc antibody incapable of recognizing this variant (Figure 2). However, the mechanism of N’Δ truncation remains unresolved. Analysis of patient-derived glioblastoma neurospheres and tumor cell lines revealed higher abundance of N’ΔGLI1 compared to the wild-type protein suggesting the absence of two inhibitory regions located in the full-length GLI1 N-terminus: an N’ degron and SUFU-binding site [138]. Similar to GLI1, N’ΔGLI1 can be phosphorylated via a p53-dependent mechanism, leading to a decrease in activity [138].

### 4.4. tGLI1

Our laboratory discovered a second novel GLI1 isoform, named truncated GLI1 (tGLI1), in 2009 [24]. Alternative splicing of the wild-type GLI1 transcript leads to an in-frame deletion of nucleotides 179–301 and amino acids 34–74, corresponding to the complete deletion of exon III and part of exon IV (Figure 2) [24]. Analysis of glioblastoma cell lines revealed that tGLI1 was not deleted at the genomic level indicating biogenesis via post-transcriptional alternative splicing. Further characterization revealed that despite the loss of 41 amino acids, tGLI1 retains all known GLI1 functional domains, responds to SHH stimulation, and translocates to the nucleus where it transactivates GLI1 target genes and other genes not regulated by GLI1 (Table 1) [24,25,28,29,30,31,32,33]. The mechanism behind tGLI1′s more potent induction of GLI1 target genes and ability to transcriptionally activate novel target genes remains unclear, but is the subject of active study. tGLI1 expression is tumor-specific: tGLI1 is frequently expressed in cancerous tissues including those of glioblastoma, breast cancer, and hepatocellular carcinoma, but not in normal adult tissues or cell lines [24,25,26,27]. tGLI1 expression distinctly differs from GLI1 and GLI1ΔN because both of these isoforms are expressed in both normal and cancer cells [23,24,139]. In Section 6, we will discuss in detail the known roles of tGLI1 in glioblastoma and breast cancer.

## 5. Aberrant SHH Signaling in Cancers

SHH signaling is tightly linked to many fundamental processes, including embryonic development and tissue homeostasis [2,3]. Emerging evidence suggests that dysregulation of this signaling axis could contribute to tumorigenesis, metastasis, and drug resistance. At the molecular level, it has been shown that SHH signaling drives the progression of cancers by regulating cancer cell proliferation, malignancy, metastasis, and the expansion of CSCs [175,176]. Aberrant pathway activation has been linked to the development of several cancer types with three distinct mechanisms proposed: ligand-independent signaling resulting from protein mutations as in the cases of BCC, medulloblastoma, and rhabdomyosarcoma by way of *PTCH1* mutations (Type I); ligand-dependent autocrine or juxtacrine signaling via overexpression of the SHH ligand observed in colorectal, ovarian, and pancreatic cancers (Type II); and ligand-dependent paracrine signaling via SHH ligand overexpression induced by tumor–stroma interactions commonly found in colon, pancreatic, and prostate cancers (Type III) [4,17,18].

### 5.1. Type I Signaling—Autonomous and SHH Ligand-Independent

Though unknown at the time, aberrant SHH signaling was first linked to cancer when Robert J. Gorlin described Gorlin Syndrome (also referred to as naevoid basal cell carcinoma syndrome or basal cell naevus syndrome) in 1960 [177]. Characterized by developmental abnormalities and distinct postnatal cancer occurrence including medulloblastoma (1–2% of cases) and rhabdomyosarcoma (0.5% of cases), Gorlin syndrome primarily results from inactivating *PTCH1* mutations on chromosome 9, leading to SMO hyperactivity [178,179,180,181,182,183]. In fact, nearly 90% of sporadic BCC and up to 33% of sporadic medulloblastomas show SHH pathway activation, caused most commonly by inactivating mutations in *PTCH1* or more rarely by activating mutations in *SMO* [184,185]. The connection between SHH signaling and cancer was bolstered when Dahmane et al. (1997) described that human sporadic BCC consistently expressed *GLI1*, but not *SHH* or *GLI3* [13]. This suggested that dysregulated GLI1 activity in basal cells induced BCC formation. In other cancers caused by ligand-independent SHH signaling, tumorigenesis can arise from activating *SMO* mutations or inactivating *SUFU* mutations [14,15,186]. Conversely, mutations impacting SUFU function in BCC are rare [187]. Similarly, *PTCH1* somatic mutations and loss of heterozygosity have been described in transitional cell carcinoma (TCC) of the bladder and esophageal squamous cell carcinoma [158,188,189,190].

### 5.2. Type II Signaling—Autocrine/Juxtacrine SHH Ligand-Dependent

A second mechanism of uncontrolled activation of SHH signaling may arise through increased secretion of SHH ligand by tumor cells into the immediate surrounding area. Since the SHH pathway is activated in a cell-autonomous manner, SHH ligand is produced by and taken up by the same or immediately surrounding tumor cells. This ligand-dependent autocrine/juxtacrine signaling has been described in several cancers including colorectal, prostate, liver, breast, ovarian, brain, and melanoma [8,18,191,192,193]. The pathogenesis of these tumors is distinct from BCC and medulloblastoma in that they do not present with any somatic mutational burden of the SHH pathway. Most of these tumors show increased expression of the SHH ligand and ectopic *PTCH1* and *GLI1* expression.

### 5.3. Type III Signaling—Paracrine SHH Ligand-Dependent

In contrast to Type I and Type II, paracrine ligand-dependent signaling co-opts the stromal cells within the tumor microenvironment to facilitate formation of a favorable tumoral niche. Paracrine SHH [3,194] signaling is critical during development and for the maintenance of various epithelial structures such as the small intestine. Analysis of several human tumor xenografts showed that the levels of SHH ligand mRNA expression in tumor cells correlated with increased *GLI1* and *PTCH1* mRNA levels in the stroma and not the tumor compartment, indicating that the SHH ligands activate SHH signaling in the surrounding stroma instead of the tumor epithelium [195]. In the absence of SHH pathway mutations, SHH ligands released by a subset of epithelial cancer, including colon, ovarian, pancreatic, and prostate, activate SHH signaling in the surrounding stroma [8,192,195,196,197,198,199,200]. The stromal cells, in turn, secrete paracrine growth factors such as vascular endothelial growth factor (VEGF), insulin-like growth factor (IGF), interleukin-6 (IL-6), Wnt, platelet-derived growth factor (PDGF), and bone morphogenetic proteins (BMP) to promote tumor growth [201]. For example, analysis of primary esophageal cancers revealed that even though the SHH transcript was localized to the tumor tissue, expression of *GLI1* and *PTCH1* was found in both the tumor and stroma [200]. Likewise, *SHH* expression was detected in the tumor epithelium of prostate cancer samples while *GLI1* expression, was upregulated in the stromal compartment, again providing evidence of paracrine ligand-dependent signaling to facilitate cancer development [199].

Interestingly, a “reverse paracrine” signaling model has been observed. In this instance, stromal SHH is believed to provide a suitable microenvironment for promoting tumor growth. Although this phenomenon has yet to be observed in solid tumors, it has been observed in several hematological malignancies including multiple myeloma, lymphoma, and leukemia [162,202]. Acute myeloid leukemia (AML) stems from a differentiation defect of hematopoietic stem and progenitor cells (HSPCs) in the bone marrow, resulting in the accumulation of immature blast cells that displace the normal hematopoietic system. Within the bone marrow microenvironment, AML blasts interact and communicate with stromal and immune cells [203]. The SHH pathway is under active study for its roles in leukemic stem cell regulation and acquired drug resistance of poor prognostic AML. SMO and GLI1 expression levels were found to correlate with acquired radio- and drug-resistance in human myeloid cell lines [204]. Patients who relapsed after ribavirin treatment presented with elevated levels of GLI1 and the UDP glucuronosyltransferase (UGT1A) which prevents ribavirin from eliciting its effect [205]. Interestingly, GLI1 was found to be sufficient to promote UGT1A expression whereas pharmacological or genetic GLI1 inhibition abrogated the acquired resistance. Furthermore, several clinical trials investigating the use of SMO inhibitors alone or in combination with compounds blocking driver mechanisms in AML have been initiated. In fact, the SMO inhibitor Glasdegib, in combination with low-dose cytarabine, was recently approved for AML treatment of elderly patients [206].

That said, findings by Becher et al. (2008) suggest that PDGF-induced gliomas may employ paracrine SHH signaling [207]. Using a GLI1-luc reporter mouse model, they were able to observe two populations of SHH-producing stromal cells located within the perivascular niche: low-cycling astrocytes and endothelial cells demonstrating an SHH-producing microenvironment. These SHH-immunoreactive astrocytes resided in the perivascular niche in close association with nestin-expressing tumor cells. Importantly, these SHH-immunoreactive cells were not derived from the cell of origin suggesting these reactive astrocytes were recruited to the tumor to provide an SHH-rich microenvironment [207].

### 5.4. Cancer Stem Cells

During tumor development, SHH signaling has three major roles: driving tumor development, promoting tumor growth, and regulating residual cancer cells after therapy [208]. Metastasis and relapse following successful treatment of primary tumors are major challenges for cancer therapies. The mechanism behind late-stage recurrence remains unknown. However, one hypothesis suggests the presence of a subset of stem-like cancer cells that possess stem cell characteristics. These cancer stem cells (CSC) have the ability to give rise to the heterogeneous cell population found within the primary tumor and are considered to be the metastasis-initiating tumor cells [209,210,211]. Multiple studies implicate the SHH pathway as a driver of CSC maintenance in cancers of the brain [191], breast [212,213], colon [214], lung [173,215], ovary [216], pancreas [217], prostate [218], as well as multiple myeloma [219] and chronic myelogenous leukemia [220,221]. GLI1 proteins regulate the expression of the pluripotency factors Nanog, octamer binding transcription factor 4 (OCT4), SOX2, WNT-2, CD44, and Kruppel-like factor 4 (KLF4) [148,165,167,168]. Recently, we have shown the novel tGLI1 isoform to promote the glioma stem cell population via upregulation of CD44 and the breast cancer stem cell population through upregulation of CD44, Nanog, OCT4, and Sox2 (Section 6) [31,33]. These data demonstrate that amplified SHH signaling is important for the self-renewal and maintenance of CSCs.

## 6. SHH Signaling and Human Cancer

**Figure 3:**
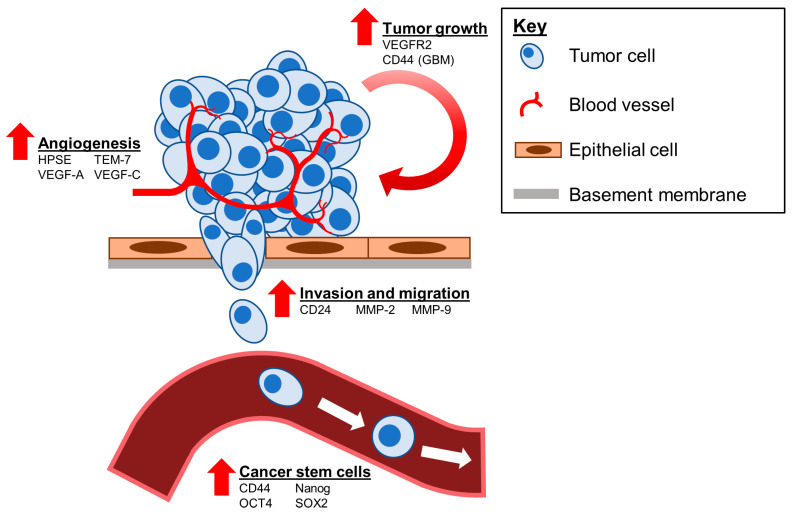
tGLI1-mediated oncogenic phenotypes in glioblastoma and breast cancer. In addition to regulating several known GLI1 target genes, tGLI1 gains the ability to modulate several potent oncogenic phenotypes via transcriptional activation of genes not regulated by GLI1: (1) angiogenesis via HPSE, TEM-7, VEGF-A, and VEGF-C; (2) tumor growth rate by increasing VEGFR2 and CD44 (GBM only) expression; (3) invasion and migration via CD24, MMP-2 (BrCa only), and MMP-9 (BrCa only); and (4) cancer stemness via upregulation of CD44, Nanog, OCT4, and SOX2. BrCa, breast cancer; GBM, glioblastoma.

Tightly controlled SHH signaling is necessary for the proper embryonic development and maintenance of differentiated tissues. However, a plethora of studies have linked dysregulated SHH signaling to numerous diseases from insufficient pathway activation, resulting in tissue degradation to aberrant signaling, leading to tumorigenesis. Genetic alterations in this pathway are commonly loss-of-function changes to suppressors of the pathway (e.g., *PTCH1* and *SUFU*) or gain-of-function changes to promotors of the pathway (e.g., SMO and GLI). Patients afflicted by Gorlin syndrome present with loss of *PTCH1* heterozygosity, increasing the likelihood of developing a distinct set of solid tumors: BCC, medulloblastoma, and rhabdomyosarcoma [177]. *PTCH1* mutations leading to SMO hyperactivity can be leveraged for therapeutic efficacy and currently represent the most effective therapeutic strategy. Indeed, the SMO inhibitors Vismodegib and Sonidegib are FDA-approved to treat SMO hyperactivity in BCC and have shown promising efficacy in SHH-dependent preclinical models of medulloblastoma and others [222,223,224,225,226,227,228,229,230,231]. However, the discovery of alternative splice variants of the GLI1 transcript, GLI1ΔN and tGLI1, suggest that these variants may constitute additional therapeutic targets or mechanisms underlying resistance to SHH inhibitors. Therefore, understanding the role of SHH dysregulation in SHH-mediated cancers and how the novel GLI1 splice variants may promote various phenotypes is paramount to further mechanistic discovery and drug development. The remainder of this review is dedicated to summarizing the pertinent functions of tGLI1-mediated SHH signaling in glioblastoma and breast cancer (Figure 3), two cancers in which tGLI1 activity has been validated, and how tGLI1 activity can be leveraged to devise novel treatments.

### 6.1. Glioblastoma

Glioblastoma (GBM) is the most common and most lethal brain tumor in adults, accounting for 15% of all brain tumors [232]. Given that the *GLI1* amplification was discovered in gliomas, it is self-evident that aberrant SHH signaling plays a central role in glioma pathogenesis and tumor progression. Despite the advances made in surgical techniques, radiotherapy and chemotherapy, GBM prognosis remains poor with a median survival of 14 months following diagnosis and less than 5% of patients surviving five years postdiagnosis [233]. Recent studies identified subpopulations of stem cell-like tumor cells, termed glioma stem cells (GSCs), that exhibit the ability to self-renew, persistently proliferate, and differentiate into multiple cell lineages, thereby acting as key drivers of tumor initiation, recurrence, and chemoresistance [234,235,236,237]. The SHH pathway has been shown to play an essential role in potentiation of the GSC function [191]. Gene expression of glioma samples revealed enrichment of a stemness signature that included the stemness markers *NANOG, OCT4, SOX2*, and *BMI1* that correlated with tumor grade. Further characterization revealed that SHH-mediated GLI1 signaling regulated the self-renewal of CD133^+^ glioma CSCs [191]. Gliomas with hyperactive SMO initially respond to SMO inhibitors. However, resistance to SMO antagonists frequently emerge due to germline or acquired *SMO* mutations [238,239,240,241,242]. This has led to investigation of orthogonal therapeutic strategies that may prove efficacious against refractory gliomas or as adjuvant therapies [243,244]. GLI1 activity in GBM is also regulated through crosstalk with other pathways including Ras, Myc, and Akt, which may contribute to resistance to SHH inhibitors in gliomas [242].

While attempting to gain further insight into the GLI1 structure, our laboratory discovered the novel tGLI1 isoform while attempting to clone GLI1 cDNA. To produce the tGLI1 variant, the *GLI1* mRNA transcript undergoes alternative splicing in which 41 amino acids are deleted, corresponding to the loss of exon III and part of exon IV [24]. This corresponds to a difference of only 4.5 kD between the high molecular weight proteins. We have optimized conditions to reliably separate two GLI1 isoforms by SDS-PAGE and validated custom-made antibodies to specifically detect tGLI1. However, the difficulty in separating the two proteins could mean that functions previously ascribed to GLI1 are actually due to tGLI1-specific activity [25,33]. Our laboratory, and others, have shown the tGLI1 variant to be expressed in most GBM specimens and patient-derived xenografts, but undetectable in normal brain cells or tissues [24,26,27,29]. Interestingly, studies by Stecca et al. (2009) did not observe tGLI1 in GBM cell lines and patient-derived neurospheres despite using two antibodies that recognize regions conserved after alternative splicing [138]. This may be due to the use of a 7.5% acrylamide gel instead of the optimized 5.5% gel that we described. We reported that tGLI1 regulates known GLI1 target genes to a similar degree as the wild-type GLI1, but gained the ability to transcriptionally activate genes not regulated by GLI1, including *CD24*, *CD44*, *HPSE*, *TEM7*, *VEGF-A*, *VEGF-C*, and *VEGFR2*, thus promoting cancer cell growth, migration, invasion, and angiogenesis [24,25,28,30,31]. tGLI1-mediated upregulation of *CD24* expression was found to increase GBM invasion [24]. CD24 is known to recruit adhesion molecules to lipid rafts, thereby contributing to tumor cell migration, dissemination, and metastasis [245,246]. Using a GBM xenograft mouse model, we showed that tGLI1-expressing tumor cells were significantly more infiltrative than GLI1-expressing cells thus promoting the aggressiveness of GBM [24]. tGLI1 also gains the ability to promote the aggressiveness of GBM as a novel mediator of angiogenesis through regulation of VEGFR2 and VEGF-A in an autocrine loop. VEGF-A binds to the VEGFR1/2 receptor, leading to downstream stimulation of endothelial cell proliferation and neovascularization [247]. Evidence from our laboratory and those of other groups suggests that tGLI1 may be regarded as a novel therapeutic target for GBM. This notion is supported by tGLI1 tumor-specific expression and that tGLI1 plays an important role in tumor growth, angiogenesis and CSC renewal in GBM.

### 6.2. Breast Cancer

Breast cancer is the second leading cause of cancer-related mortality in women and metastasis to distant organs result in 90% of breast cancer deaths [248]. Metastatic breast cancers occur in 20–30% of cases with a 5 year survival rate of 22% [249]. Dysregulation of the SHH pathway is implicated in the development and proliferation of breast cancer [250,251]. Although improper SHH signaling can arise from a variety of mechanisms (see Section 5 above), aberrant SHH signaling in breast cancer tends to occur through Type II ligand-dependent signaling in which tumors cells profusely secrete active SHH ligand into the immediate surrounding area, leading to self-activation or activation of juxtaposed tumor cells [193,252]. This is supported by findings that *SHH*, *PTCH1*, and *GLI1* mutations are exceedingly rare in breast cancer which argues against the potential involvement of mutational activation of the SHH pathway in breast cancer [253,254,255]. Nevertheless, expression of SHH pathway components correlates with more aggressive breast cancer subtypes and predicts poor overall survival [256,257]. Additionally, basal expression of GLI1 and GLI2 is higher in triple negative breast cancer (TNBC) than in hormone-receptor (HR) positive breast cancer suggesting this pathway may be especially relevant to this subtype [258]. GLI1 knockdown in HR-negative breast cancer cells led to reduced viability in vitro [259].

Over the past decade, our lab has observed tGLI1 to be an important player in breast cancer progression and metastasis. Similar to GBM, tGLI1 is expressed in a tumor specific pattern, i.e., observed only in malignant breast tissue and cells, and transactivates novel target genes not regulated by GLI1 to promote aggressive phenotypes [24,25,28,30,31]. Separate from GLI1, we observed tGLI1-mediated upregulation of matrix metalloproteinase-2 (MMP-2) and MMP-9 which facilitate breast cancer invasion through degradation of the extracellular matrix [139]. Additionally, we made the novel observation that tGLI1 physically and functionally interacts with signal transducer and activator of transcription 3 (STAT3) in human epidermal growth factor receptor 2 (HER2)-positive and TNBC to promote aggressiveness [32]. STAT3 is an oncogenic transcription factor that is downstream of Janus-activated kinase 2 (JAK2), a non-receptor tyrosine kinase commonly amplified and hyperactive in TNBC and HER2-enriched breast cancers [260,261,262]. We identified and validated three genes that are directly activated by STAT3-GLI1 and/or STAT3-tGLI1 cooperation, namely, *R-Ras2*, *Cep70*, and *UPF3A* (Table 1) [32]. Moreover, the observed co-activation of JAK2-STAT3 and SHH signaling pathways suggests that combination of JAK2 and SMO inhibitors may synergize against TNBC and HER2-enriched breast cancers. Recently, we have gleaned evidence that tGLI1 is intimately involved in breast cancer metastasis brain tropism [33]. Using an intracardiac mouse model, we found that tGLI1 expression increased the formation of breast cancer brain metastases without impacting bone metastases. Conversely, tGLI1 knockdown abrogated this effect. GLI1 expression did not increase brain metastasis formation. Immunohistochemical analysis revealed that tGLI1 expression is increased in lymph node metastases and breast cancer brain metastasis (BCBM) samples compared to unmatched primary breast carcinomas suggesting tGLI1-positive breast cancer may have increased metastatic potential [33]. To understand tGLI1-mediated metastasis, we assessed the effect of tGLI1 expression on the CSC population which are considered the metastasis-initiating cells [210]. SHH pathway activation may be especially relevant to the viability of the CD44+/CD24--expressing breast CSCs, which are believed to be particularly chemoresistant due to their relative quiescence and expression of drug efflux pumps [263]. We also demonstrated that tGLI1-positive CSCs strongly activate and interact with astrocytes in vitro and tGLI1-positive BCBM tumors activate astrocytes in vivo [33]. Reactive astrocytes have been shown to support the arrest, extravasation, and growth of breast cancer cells in the brain [264]. Clearly, there is substantial evidence calling for reassessment of the *GLI1* gene and the importance of alternative splicing in the SHH signaling pathway.

### 6.3. Therapeutic Targets Regulated by tGLI1

The prevalence of SHH-driven tumors demonstrates the potential therapeutic utility of hedgehog pathway inhibitors. However, the clinical efficacy of SMO-targeting therapies has been mixed, while only a single GLI1-targeting agent (arsenic trioxide) has been approved by the FDA [19,265]. There is an obvious need to further understand the biology of tumors with aberrant SMO and GLI1 signaling, and identify novel actionable targets. Given its tumor-specific expression and potent oncogenic effects, tGLI1 is an ideal therapeutic target [24,25,28,30,31].

Currently, no tGLI1 inhibitors have been developed to date. However, several tGLI1 target genes can be targeted using previously developed agents including HPSE, VEGF-A, and VEGFR2. Antiangiogenic therapies are an attractive focus for cancer drug development because heightened tumor vascularization is canonical phenotype of aggressive cancers [266]. HPSE has been extensively studied as a potential anti-cancer drug target [267,268]. Heparanase is an endoglucuronidase responsible for heparan sulfate cleavage and regulates the structure and function of heparan sulfate proteoglycans, leading to disassembly of the extracellular matrix [267]. Several studies have validated the significance of HPSE as a valid anti-cancer drug target by demonstrating inhibition of human myeloma, lymphoma, glioma, sarcoma, mesothelioma and pancreatic tumor growth in mice treated with the heparin-like heparanase-inhibiting compounds Roneparstat and Pixatimod [269]. Roneparstat was well-tolerated by patients with relapsed/refractory multiple myeoloma up to 400 mg daily (NCT01764880) [270]. Of the 17 patients enrolled, one presented with partial disease response, 9 with stable disease, with the remaining 7 experiencing disease progression over the course of the trial. HPSE inhibition is not expected to cause direct tumor cell kill, and, as expected, this study did not provide evidence of a potential direct anti-myeloma effect of ronepartstat in humans. However, the favorable safety profile of roneparstat implies its combination with other anti-cancer compounds is the next step in this field [271].

VEGF-A and VEGFRs are well-characterized targets for antiangiogenic therapeutics and these drugs have shown a varying range of effectiveness across tumor types [272]. Monoclonal antibodies such as Bevacizumab (Avastin), a humanized monoclonal antibody that blocks VEGF-A, or small molecules such as vandetanib, which inhibits tyrosine kinases downstream of VEGF, are examples of anti-VEGF treatment. A Phase 1 trial of Bevacizumab showed it was well tolerated with a favorable pharmacokinetic profile [273]. It is currently FDA-approved for metastatic colorectal cancer, non-small-cell lung cancer, glioblastoma, renal cell carcinoma, epithelial ovarian cancer, fallopian tube cancer, primary peritoneal cancer, and cervical cancer [274]. Unfortunately, bevacizumab failed to increase survival among patients with breast, pancreatic, and prostate cancers and melanoma [275]. Afilbercept (Zaltrap) is another immunotherapy that binds VEGF-A precluding its interaction with VEGFR1/2 [276]. Afilbercept combined with chemotherapy is currently a second-line therapy for metastatic colorectal cancer patients. The final monoclonal antibody targeting the VEGF-VEGFR interaction is ramucirumab (Cyramza), a VEGFR2 monoclonal antibody approved for the treatment of metastatic colorectal cancer, non-small-cell lung cancer, and gastroesophageal junction adenocarcinoma [277]. In the second-line setting, ramucirumab significantly increased overall survival of patients with unresectable gastroesophageal tumors who had progressed on first-line chemotherapy regimens (5.2 vs. 3.8 months; *p* = 0.047). When combined with paclitaxel, ramucirumab significantly increased overall and progression-free survival in patients with metastatic gastric cancer [278]. In our efforts to delineate the function of tGLI1, we have discovered that tGLI1 is able to increase not only VEGF-A expression, but also VEGFR2, leading to a powerful autocrine loop [28].

In addition to inhibiting tGLI1 target genes, inhibition of tGLI1 synthesis and transcriptional co-regulators could lead to tumor cell kill in tGLI1-high tumors. However, further studies are needed to delineate the splicing mechanisms responsible for the in-frame deletion of the wild-type GLI1 gene transcript that results in tGLI1 synthesis. Furthermore, whether tGLI1 requires transcription co-factors to activate target gene expression remains unknown. TGLI1 may also be subject to regulation by noncanonical pathways. Addressing these knowledge gaps will aid development of novel strategies to target tGLI1-driven tumors.

## 7. Conclusions and Future Directions

The SHH pathway is evolutionarily conserved and essential for the regulation of normal development and differentiation in vertebrates. However, aberrant SHH pathway activation by either mutation or ligand overexpression is closely correlated with several cancers. Although the link between the SHH signaling pathway and tumorigenesis varies by cancer type, it is clear that the aberrant activation of SHH signaling leads to the growth, proliferation, and invasion of tumor cells. Furthermore, data indicate that the GLI transcription factors are regulated by several oncogenic signaling pathways in addition to canonical PTCH-SMO signaling. Thus, targeting the SHH pathway may provide therapeutic options. In fact, the truncated GLI1 (tGLI1) splice variant presents a unique opportunity to validate a novel actionable target for gliomas and breast cancer brain metastases due to its tumor-specific expression and potent oncogenic effects. The alternative splicing of the *GLI1* transcript creates a more potent oncogenic protein. Going forward, it will be important to determine the precise mechanisms by which tGLI1 expression is regulated including the splicing machinery that influences tGLI1 expression levels. Additionally, whether tGLI1 is regulated by any noncanonical pathways should be investigated. Such interactions could allow tGLI1 to serve as an oncogenic signaling hub similar to STAT3 that permits convergence of several signaling pathways to create a novel mechanism of tumor progression. Finally, elucidating the mechanisms by which tGLI1 activates its target genes is critical to identifying other novel targets of tGLI1 that could contribute to the aggressive phenotypes of tGLI1-positive tumors. Given the established role of aberrant SHH signaling in tumorigenesis of several human cancers, understanding the fundamental mechanisms of SHH pathway regulation and the impact of alternative GLI1 splice variants will aid the development of novel therapeutics and achieve improved therapeutic outcomes for these patients.

## Figures and Tables

**Table 1:** Known target genes of GLI1 isoforms in human cancers.

Isoform	Target Gene	Cancer	Reference
GLI1	ABCG2	Diffuse large B-cell lymphoma	[140]
	ANO1	Pancreatic ductal adenocarcinoma	[141]
	AQP1	Glioma	[142]
	Bcl-2	Colorectal adenocarcinoma, basal cell carcinoma, B-cell chronic lymphocytic leukemia	[143,144,145]
	BHLHE41	Pancreatic ductal adenocarcinoma	[146]
	Bmi1	Medulloblastoma	[147]
	c-Myc	Pancreatic adenocarcinoma	[148]
	CXCR4	Breast cancer, pancreatic ductal adenocarcinoma	[149,150]
	Cyclin D1	Pancreatic ductal adenocarcinoma	[151]
	Cyclin D2	Rhabdomyosarcoma, medulloblastoma, astrocytoma, cervical cancer	[152,153,154]
	DMNT1	Pancreatic ductal adenocarcinoma	[155]
	ENC1	Medulloblastoma, rhabdomyosarcoma	[156]
	E2F1	Melanoma	[157]
	FOXM1	Basal cell carcinoma, bladder cancer, glioblastoma	[158,159,160]
	FOXS1	Medulloblastoma, rhabdomyosarcoma	[156]
	GLI1	B-cell chronic lymphocytic leukemia, chronic myelogeneous leukemia, medulloblastoma, multiple myeloma, rhabdomyosarcoma	[145,156,161,162]
	GLI2	B-cell chronic lymphocytic leukemia, multiple myeloma	[145,162]
	H19	Bladder cancer	[158]
	HHIP	Medulloblastoma, rhabdomyosarcoma	[163]
	IGF2	Bladder cancer	[158]
	IGFBP-6	Rhabdomyosarcoma, neuroblastoma, colon cancer	[164]
	IL-7	Pancreatic ductal carcinoma	[151]
	KLF4	Colon cancer	[165]
	Krox-20	Medulloblastoma, cervical cancer	[153]
	MUC5AC	Pancreatic ductal adenocarcinoma	[166]
	Nanog	Colon cancer, glioblastoma, medulloblastoma, pancreatic adenocarcinoma	[148,165,167,168]
	NEO1	Basal cell carcinoma, medulloblastoma	[169,170]
	NKX2.2	Medulloblastoma, astrocytoma	[154]
	OCT4	Colon cancer, pancreatic adenocarcinoma	[148,165]
	Osteopontin	Melanoma	[164]
	PAX6	Medulloblastoma, astrocytoma	[154]
	PDGFRα	Basal cell carcinoma	[171]
	Plakoglobin	Rhabdomyosarcoma, medulloblastoma, astrocytoma	[152,154]
	PLAT	Medulloblastoma, rhabdomyosarcoma	[156]
	PRPSAP1	Cervical cancer	[153]
	PSF2	Bladder cancer	[158]
	PTCH1	Chronic myelogeneous leukemia, medulloblastoma, multiple myeloma, rhabdomyosarcoma	[154,156,161,162]
	PTCH2	Medulloblastoma, rhabdomyosarcoma	[156]
	SHH	Chronic myelogeneous leukemia	[161]
	SMO	Chronic myelogeneous leukemia	[161]
	Snail1	Melanoma	[172]
	SOSTDC1	Medulloblastoma, rhabdomyosarcoma	[156]
	Sox2	Colon cancer, non-small-cell lung cancer, pancreatic ductal adenocarcinoma	[148,165,173]
	SPP1	Bladder, melanoma	[158]
	Twist1	Melanoma	[172]
	WNT-2	Colon cancer	[165]
	Zeb1	Melanoma	[172]
tGLI1	CD24	Breast, glioblastoma	[24,139]
	CD44	Breast, glioblastoma	[31]
	Cep70 ^1^	Breast cancer	[32]
	HPSE	Glioblastoma	[29]
	MMP-2	Breast cancer	[139]
	MMP-9	Breast cancer	[139]
	Nanog	Breast cancer	[33]
	OCT4	Breast cancer	[33]
	PTCH1	Glioblastoma	[24]
	R-Ras2 ^1^	Breast cancer	[32]
	Sox2	Breast cancer	[33]
	TEM7	Glioblastoma	[30]
	UPF3A ^1^	Breast cancer	[32]
	VEGF-A	Breast, glioblastoma	[139,174]
	VEGF-C	Glioblastoma	[30]
	VEGFR2	Breast, medulloblastoma	[28]

^1^ tGLI1 must be complexed with STAT3.
